# Ocular tolerability and efficacy of intravitreal and subconjunctival injections of sirolimus in patients with non-infectious uveitis: primary 6-month results of the SAVE Study

**DOI:** 10.1186/1869-5760-3-32

**Published:** 2013-02-11

**Authors:** Quan Dong Nguyen, Mohamed A Ibrahim, Anthony Watters, Millena Bittencourt, Jithin Yohannan, Yasir J Sepah, James P Dunn, Joel Naor, Naveed Shams, Ovais Shaikh, Henry Alexander Leder, Diana V Do

**Affiliations:** 1 Wilmer Eye Institute, Johns Hopkins University School of Medicine, 600 North Wolfe Street, Maumenee 745, Baltimore, MD, USA; 2 Santen Pharmaceuticals, Inc., Emeryville, CA, USA; 3 Santen Pharmaceuticals, Osaka, Japan; 4 Stanley M. Truhlsen Eye Institute, University of Nebraska Medical Center, 3902 Leavenworth Street, Omaha, NE, USA

**Keywords:** Sirolimus, mTOR, Uveitis, Intravitreal, Subconjunctival

## Abstract

**Background:**

The purpose of this study is to evaluate the ocular tolerability and efficacy of sirolimus administered as subconjunctival or intravitreal injections in patients with non-infectious uveitis. Sirolimus as a Therapeutic Approach for Uveitis (SAVE) is a prospective, randomized, open-label, interventional study. Thirty patients were enrolled and randomized in 1:1 ratio to receive either intravitreal injections of 352 μg sirolimus or subconjunctival injections of 1,320 μg at days 0, 60, and 120, with primary endpoint at month 6.

**Results:**

At month 6, all subjects with active uveitis at baseline showed reduction in vitreous haze of one or more steps. Forty percent of subjects showed reduction of two steps or more of vitreous haze (four in each group), and 60% showed a reduction of one-step vitreous haze (seven in group 1 and five in group 2). Changes in the inflammatory indices were statistically significant (*p* < 0.05) in both study groups. Thirty percent of patients gained one or more lines of visual acuity, 20% lost one or more lines, and 50% maintained the same visual acuity. There were no statistically significant differences between the two study groups at month 6. No serious adverse events were found to be related to the study drug.

**Conclusion:**

Local administration of sirolimus, either intravitreally or subconjunctivally, appears to be safe and tolerable. No drug-related systemic adverse events or serious adverse events were noted. Sirolimus delivered as either an intravitreal or subconjunctival injection has demonstrated bioactivity as an immunomodulatory and corticosteroid-sparing agent in reducing vitreous haze and cells, improving visual acuity, and in decreasing the need for systemic corticosteroids.

## Background

Non-infectious uveitis is often of a putative autoimmune nature, can affect patients of different age groups, and can be limited to the eye or be part of a systemic syndrome. Posterior uveitis, intermediate uveitis, and panuveitis are less common than anterior uveitis, which constitutes 60% to 75% of all uveitis and may be chronic and recurrent in up to two-third of the cases [1-3]. However, posterior uveitis correlates more frequently with irreversible visual impairment and is more challenging to manage. The triggers of the vigorous immunologic and inflammatory responses against ocular antigens are poorly understood, and the mechanism by which the immune privilege is lost is yet to be fully described. Indeed, it is well known that the ocular autoimmune activity can be driven by lymphocytes in either the Th1 or Th17 response [4]. The Th1 response has been related with R14-specific T cells and with more relapsing disease than seen in the Th17 response [4].

The primary goal in the management of non-infectious uveitis is to suppress inflammation and achieve remission [5,6]. Since its first use in 1951, corticosteroids (CS) have been the first line of treatment for non-infectious uveitis and are the only class of drugs approved by the Food and Drug Administration (FDA) to treat such pathology [7]. Both systemic and the local forms of CS are used to treat posterior uveitis; not all patients, however, can tolerate their side effects. Furthermore, satisfactory control may not be achievable in some cases, even with the correct use and employment of high doses of CS [7].

Immunomodulatory therapy (IMT) has become not only a good alternative to control the inflammatory process but also an adjunctive therapy that aids the reduction of CS burden and their complications. While CS is usually required to control acute inflammation, IMT agents are needed to downregulate chronic inflammation and prevent recurrences [8]. Drugs that primarily target T-cells, like cyclosporine and tacrolimus, have demonstrated efficacy when employed in the treatment of uveitis [9-13]. IMT has been employed to avoid further sequelae such as cataract, glaucoma, proliferative vitreoretinopathy, cystoid macular edema, vascular occlusion, and blindness [14].

Sirolimus, also known as rapamycin, was isolated in the 1970s from *Streptomyces hygroscopicus* in soil samples from Easter Island [15]. Sirolimus is an immunosuppressant that works through inhibition of the mammalian target of rapamycin (mTOR) by binding to the immunophilin FK protein 12 (FKBP-12) [15], and thus interrupts the inflammatory cascade that leads to T-cell activation and proliferation. It also suppresses T-cell proliferation through the inhibition of IL-2, IL-4, and IL-15 employing calcium (Ca^2+^)-dependent or Ca^2+^-independent pathways [16,17].

Owing to its unique mechanism of action and favorable side effect profile, sirolimus has been increasingly proposed as an alternative immunosuppressant in organ transplantation. Sirolimus is the active ingredient in two FDA-approved products, specifically Rapamune®, an immunosuppressive agent used in renal transplant patients, and CYPHER® Sirolimus-eluting Coronary Stent approved for improving coronary luminal diameter in patients with symptomatic ischemic disease. In order to allow higher target tissue levels and reduce systemic exposure, a proprietary local formulation of sirolimus was developed that, based on preclinical animal toxicity and pharmacokinetic studies, is amenable to both intraocular (intravitreal (IVT)) and extraocular (subconjunctival (SCJ)) injection. When administered by SCJ injection, a drug depot is formed that subsequently dissolves slowly and diffuses across sclera based on the physicochemical properties of sirolimus [18]. Blood levels of sirolimus after SCJ administration peaks on day 0 to dose-dependent levels: 3.62 ng/ml for a dose of 440 μg and 9.32 ng/ml for a dose of 1,320 μg [18]. By day 7, sirolimus blood levels decrease to less than 3 ng/ml and subsequently become minimally quantifiable, if at all, by day 14 and beyond [18]. Following intravitreal administration, the formulation forms a non-dispersive depot in the vitreous and localizes in the inferior portion of the vitreous humor. The depot subsequently dissolves slowly, and sirolimus diffuses through the vitreous humor to other ocular layers with the highest concentration in the vitreous followed by the retina and choroid and the lowest concentration in the sclera and blood with detectable ocular tissue levels extending for 60 days after single intravitreal administration [19]. After intravitreal administration of 352 μg, sirolimus blood levels peak to <2 ng/ml by the second day and decreases subsequently over the following days [18,19] with half-life of 8 to 9 days [19]. It is also important to recognize that the lowest therapeutic levels of sirolimus in organ transplant and cardiac patients are 5 to 15 ng/ml [19]. Based on the current knowledge of sirolimus and its potential anti-inflammatory effect, we set forth to evaluate the potential role of locally administered sirolimus in non-infectious uveitis.

## Results

### Demographics and baseline characteristics

Thirty patients with a mean age of 47 (±18.8) years were enrolled in the study. At screening, 23 of the study participants (73%) had active uveitis, of whom 8 subjects (23%) were *not* receiving any medication to control uveitis (disease category 1) and 15 subjects (50%) were receiving prednisone ≥10 mg/day (disease category 2). Seven subjects (27%), including two with punctate inner choroidopathy, had inactive uveitis at baseline and were receiving prednisone <10 mg/day and/or other immunosuppressant (disease category 3). At baseline, 10 study eyes (33%) were pseudophakic, 4 eyes had clear lens (13%), and 16 eyes had a pre-existing cataract (53%). The average intraocular pressure (IOP) in the study eyes at baseline was 14.7 mmHg (±3.4). Three patients (10%) had a history of bilateral glaucoma at baseline controlled with medication; one patient had a history of bilateral trabeculectomy. Baseline demographics and disease characteristics among study groups are summarized in Table 1.

**Table 1:** Demographics and baseline characteristics of study participants

	**Total (*n *= 30)**	**Intravitreal (*n = *15)**	**Subconjunctival (*n = *15)**
Gender (% (*n*))			
Male	50 (15)	60 (9)	40 (6)
Female	50 (15)	40 (6)	60 (9)
Age (year (*±*SD))	47 (±18.8)	45 (±19.8)	48 (±18.2)
Race (% (*n*))			
Caucasian	77 (23)	73 (11)	80 (12)
African/American	20 (6)	20 (3)	20 (3)
Others	3 (1)	7 (1)	-
Disease category (% (*n*))			
Category 1: active without treatment	23 (7)	20 (3)	27 (4)
Category 2: active with treatment	50 (15)	60 (9)	40 (6)
Category 3: inactive with treatment	27 (8)	20 (3)	33 (5)
Anatomical location (% (*n*))			
Intermediate	30 (9)	33 (5)	27 (4)
Posterior	60 (18)	60 (9)	60 (9)
Panuveitis	10 (3)	7 (1)	13 (2)
Underlying disease (% (*n*))			
Birdshot choroidopathy	13 (4)	7 (1)	20 (3)
Sarcoidosis	13 (4)	-	27 (4)
Punctate inner choroidopathy	7 (2)	7 (1)	7 (1)
Multifocal choroiditis	7 (2)	13 (2)	-
Vogt-Koyanagi-Harada	3 (1)	7 (1)	-
Idiopathic	57 (17)	67 (10)	47 (7)
Macular thickness (CMT)			
Macular edema (% (*n*))	37 (11)	47 (7)	27 (4)
Central macular thickness (mean ± SD)	356 ± 149	377 ± 178	334 ± 116
CMT in patients without ME (mean ± SD)	269 ± 28	257 ± 31.6	278 ± 22.5
CMT in patients with ME (mean ± SD)	505 ± 156	515 ± 176	488 ± 134
Corticosteroid use (% (*n*))	67 (22)	80 (12)	67 (10)
Corticosteroid dose (mg/day)			
Category 1 (mean ± SD)	NA	NA	NA
Category 2 (mean ± SD)	28.2 ± 16.2	28.3 ± 18	27.9 ± 14.9
Category 3 (mean ± SD)	7.1 ± 3.0	7.3 ± 2.1	7.0 ± 4.0
Prior IMT use (% (*n*))	25 (7)	20 (3)	27 (4)
VA in ETDRS score (Snellen equivalent)			
Category 1 (mean ± SD)	62 ± 13 (20/63)	55 ± 6.2 (20/80)	68 ± 15.4 (20/40)
Category 2 (mean ± SD)	70 ± 17 (20/40)	66 ± 16.8 (20/50)	75 ± 18.9 (20/32)
Category 3 (mean ± SD)	72 ± 23 (20/40)	66 ± 23.1 (20/50)	75 ± 24.2 (20/32)

n, number; SD, standard deviation; CMT, central macular thickness; NA, not applicable; VA, visual acuity; ME, macular edema.

### Outcomes at the primary endpoint (month 6)

Two subjects, one from each study group and both from category 2, exited the study prior to the primary endpoint at month 6. The first patient was lost to follow-up after cataract surgery in the study eye that was performed after receiving the second scheduled dose of sirolimus. The second patient was lost to follow-up following the first injection of sirolimus for personal reasons and returned at month 6. The bioactivity data collected from both subjects were not carried forward to month 6, as there were significant amount of missing information to allow appropriate assessment; baseline data from these two subjects were removed when comparing the outcome at month 6 to baseline. Adverse events from both subjects, however, were included in the analysis of safety outcome.

#### *Safety outcome*

**Intravitreal injections** Prior to the primary endpoint at month 6, the study eyes of group 1 received 42 intravitreal injections of sirolimus, and the fellow eyes (from nine patients) received 20 intravitreal injections, raising the number of injections in this group to a total of 62 injections. The adverse events encountered with intravitreal injections of sirolimus were rare and scattered. The most commonly reported adverse event in this group was vitreous floaters. Other ocular adverse events included single instances of changed refraction resulting in blurred vision, post-injection subconjunctival hemorrhage, forehead rash (above the study eye), ocular pain and redness, and progression of pre-existing cataract and glaucoma that required combined cataract extraction and glaucoma surgery with Ahmed valve implantation in the study eye. The ocular pain, redness, and subconjunctival hemorrhage were all considered related to the injection procedure rather than sirolimus. No other ocular adverse event was considered related to either the injection procedure or the study drug. Systemic adverse events included one event each of upper respiratory infection, tooth abscess, and sciatic pain secondary to disc prolapse. No systemic adverse event was considered related to the study drug. All systemic and ocular adverse events in this group were mild to moderate in severity and resolved without sequelae.

The average IOP in the study eyes was 15.5 mmHg (±3.3) at baseline, 14.7 mmHg (±3.8) at day 60, 15 mmHg (±3.9) at day 120, and 13.1 (±2.9) at month 6. With the exception of one patient, all other study subjects had IOP <25 mmHg throughout the study.

Serious ocular adverse events included the development of rapidly progressing cataract in a study eye that resulted in a loss of visual acuity ≥6 Early Treatment Diabetic Retinopathy Study (ETDRS) lines and in cataract extraction surgery prior to day 90. The patient was lost to follow-up after the surgery and dropped out of the study. Serious adverse events also included an instance of loss of visual acuity ≥6 ETDRS lines in a fellow eye because of persistent elevation of IOP ≥35 mmHg for more than 2 weeks. In this particular patient, the fellow eye did not meet the inclusion criteria of the Sirolimus as a Therapeutic Approach for Uveitis (SAVE) Study initially because of elevated IOP (36 mmHg) despite maximal medical control. During the course of the study, the patient refused to receive the standard of care with local therapy for the fellow eye and hence sirolimus was provided to this eye when the IOP was controlled to <25 mmHg. Following day 74, recurrence of ocular hypertension and worsening of the cataract resulted in a loss of ≥6 lines of visual acuity. Combined cataract extraction and glaucoma surgery with Ahmed valve was done electively, and prednisone was increased postoperatively to the baseline level (30 mg/day). The former case of ocular serious adverse events (SAEs), with rapidly progressing cataract, was considered possibly related to the study drug, although traumatic cataract secondary to the injection procedure was also entertained as a possibility. The latter SAE with ocular hypertension requiring glaucoma surgery in the fellow eye was considered unlikely related to the study drug and more likely related to the course of the disease that may have led to worsening of the uveitic glaucoma.

**Figure 1:**
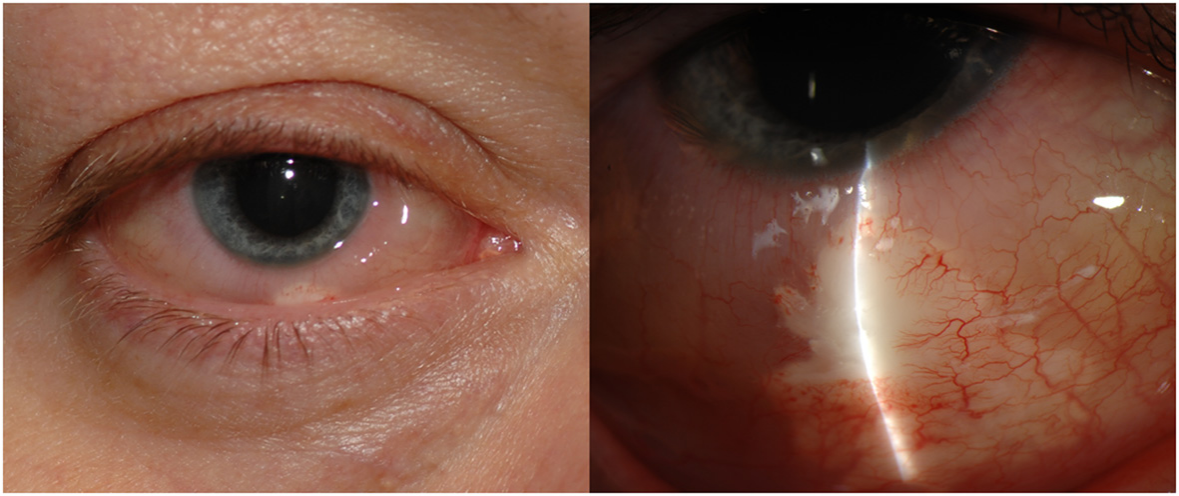
**Color slit-lamp photographs of a patient with conjunctival inflammation following subconjunctival injection of sirolimus.** Significant chemosis along with conjunctival hyperemia overlying the whitish subconjunctival aggregate of the study drug can be seen.

**Subconjunctival injections** Subjects enrolled in group 2 received a total number of 66 subconjunctival injections of sirolimus prior to month 6, 44 to the study eyes and 22 to the fellow eyes (from ten patients). The most commonly encountered adverse events were inflammation at the injection site; seven instances (10%) in six patients (40%)*.* The inflammation manifested as ocular pain and localized tenderness and hyperemia overlying the subconjunctival aggregate of sirolimus (Figure 1). The inflammations were mild to moderate, peaked at 2 weeks after injection, resolved spontaneously without sequelae within additional 2 weeks, and were considered likely related to the study drug. Other ocular adverse events included vitreous floaters (two instances in the same patient) and single instances of transient loss of vision in a study eye for about 60 min and of progression of a pre-existing cataract in a fellow eye, which required cataract surgery. Average IOP in the study eyes was 13.8 mmHg (±3.3) at baseline, 13.8 mmHg (±3.9) at day 60, 13.6 mmHg (±3.2) at day 120, and 15.3 (±4.6) at month 6. None of the study participants in this group had an IOP >25 mmHg throughout the study.

Systemic adverse events included single instances of back pain, broken wrist secondary to fall, upper respiratory and urinary tract infections, a probable diagnosis of Crohn's disease on colonoscopy, and vascular surgery of lower extremity secondary to complications of diabetes mellitus. With the exception of the injection site inflammation, all ocular and systemic adverse events were mild to moderate in nature, and none was considered to be related to the study drug. No serious adverse events were observed in this group of patients. None of the study participants, in either injection group, met any of the rescue criteria prior to the primary endpoint at month 6. Therefore, no study subjects received rescue treatment.

#### *Bioactivity outcome*

**Changes in the inflammatory indices** Summary of the inflammatory indices at baseline and the changes at months 3 and 6 are shown in Table 2.

**Table 2:** Changes from baseline in inflammatory indices, dose of corticosteroids, and visual acuity

	**All (*n *= 28)**			**Group 1 (*n *= 14)**			**Group 2 (*n *= 14)**		
	**Baseline**	**Month 3**	**Month 6**	**Baseline**	**Month 3**	**Month 6**	**Baseline**	**Month 3**	**Month 6**
Anterior chamber cells (number of patients)									
None	24	26	27	11	13	14	13	13	13
0.5+	3	2	1	2	1	-	1	1	1
1+	1	-	-	1	-	-	-	-	-
2+	-	-	-	-	-	-	-	-	-
3+	-	-	-	-	-	-	-	-	-
4+	-	-	-	-	-	-	-	-	-
Vitreous cells (number of patients)									
None	14	22	27	8	11	11	6	11	11
0.5+	5	5	1	3	3	2	2	2	1
1+	7	1	-	3	-	1	4	1	2
2+	2	-	-	-	-	-	2	-	-
3+	-	-	-	-	-	-	-	-	-
4+	-	-	-	-	-	-	-	-	-
Vitreous haze (number of patients)									
None	-	13	7	-	6	3	-	7	4
0.5+	8	11	16	3	6	9	5	5	7
1+	11	4	5	7	2	2	4	2	3
2+	8	-	-	3	-	-	5	-	-
3+	1	-	-	1	-	-	-	-	-
4+	-	-	-	-	-	-	-	-	-
Corticosteroids dose (median mg/day ± SD)									
Category 1 (*n* = 7)	NA	NA	NA	NA	NA	NA	NA	NA	NA
Category 2 (*n* = 13)	20 ± 15.7	12.5 ± 6.8	8 ± 5.7	25 ± 18.1	13.8 ± 8	7.8 ± 7.1	20 ± 11.4	12.5 ± 4.5	8.0 ± 1.9
Category 3 (*n* = 7)	9 ± 3.1	4 ± 2.9	3 ± 2.2	8.0 ± 2.1	4.0 ± 2.5	3.0 ± 1.8	9.0 ± 4.0	5.5 ± 3.5	3.0 ± 2.6
Visual acuity (mean ±SD)									
Category 1	62 ± 13.3	66 ± 13.1	61 ± 14.5	55 ± 6.2	59 ± 12.7	51 ± 10.6	68 ± 15.4	72 ± 11.5	69 ± 12.8
Category 2	71 ± 16.8	72 ± 16.3	72 ± 18.1	69 ± 15.3	70 ± 14.4	69 ± 16.9	73 ± 20.6	77 ± 20.1	76 ± 21.3
Category 3	72 ± 22.6	74 ± 18.3	74 ± 15.8	66 ± 23.1	71 ± 16.3	69 ± 14	75 ± 24.2	77 ± 21	76 ± 17.8

n, number; SD, standard deviation; NA, not applicable.

**Study categories 1 and 2 (active uveitis at baseline, n = 20)** At month 3, 12 subjects (60%) showed a reduction of two steps or more of vitreous haze (six in each group), and 8 subjects (40%) showed either no change or a reduction less than two steps (five in group 1 and three in group 2)*.* At month 6, 8 subjects (40%) showed a reduction of two steps or more of vitreous haze (four in each group), and 12 subjects (60%) showed either no change or a reduction of one-step vitreous haze (seven in group 1 and five in group 2). No patient in either category showed increase of vitreous haze of one or more steps at either month 3 or month 6.

The reduction in vitreous haze at month 3 and month 6 was statistically significant, when assessed using Wilcoxon signed rank test with *p* < 0.05, in both treatment groups and at both time points. The difference in the vitreous haze outcome between treatment groups (intravitreal vs subconjunctival) was not statistically significant, either at month 3 or month 6 (*p* = 0.901 and 0.727, respectively), when assessed by Mann–Whitney *U* test.

Comparing the outcome of both categories at month 3, 71% of subjects in category 1 (5/7, two in group 1 and three in group 2) showed a reduction of ≥2 steps of vitreous haze compared to 54% in category 2 (7/13, four in group 1 and three in group 2). Meanwhile, 29% of subjects in category 1 (2/7, one in each group) showed either no change or a reduction <2 steps compared to 46% in category 2 (6/13, four in group 1 and two in group 2).

Comparing both categories at month 6, 57% of subjects in category 1 (4/7, two in each group) showed a reduction of ≥2 steps of vitreous haze compared to 31% in category 2 (4/13, two in each group). Meanwhile, 43% of subjects in category 1 (3/7, one in group 1 and two in group 2) showed either no change or a reduction <2 steps compared to 69% in category 2 (9/13, six in group 1 and three in group 2).

**Study category 3 (inactive uveitis at baseline, n = 8)** Patients in category 3 did not show statistically significant changes in vitreous haze in any of the study groups at either month 3 or month 6 (*p* = 0.317 at month 6, Wilcoxon signed rank test).

**Figure 2:**
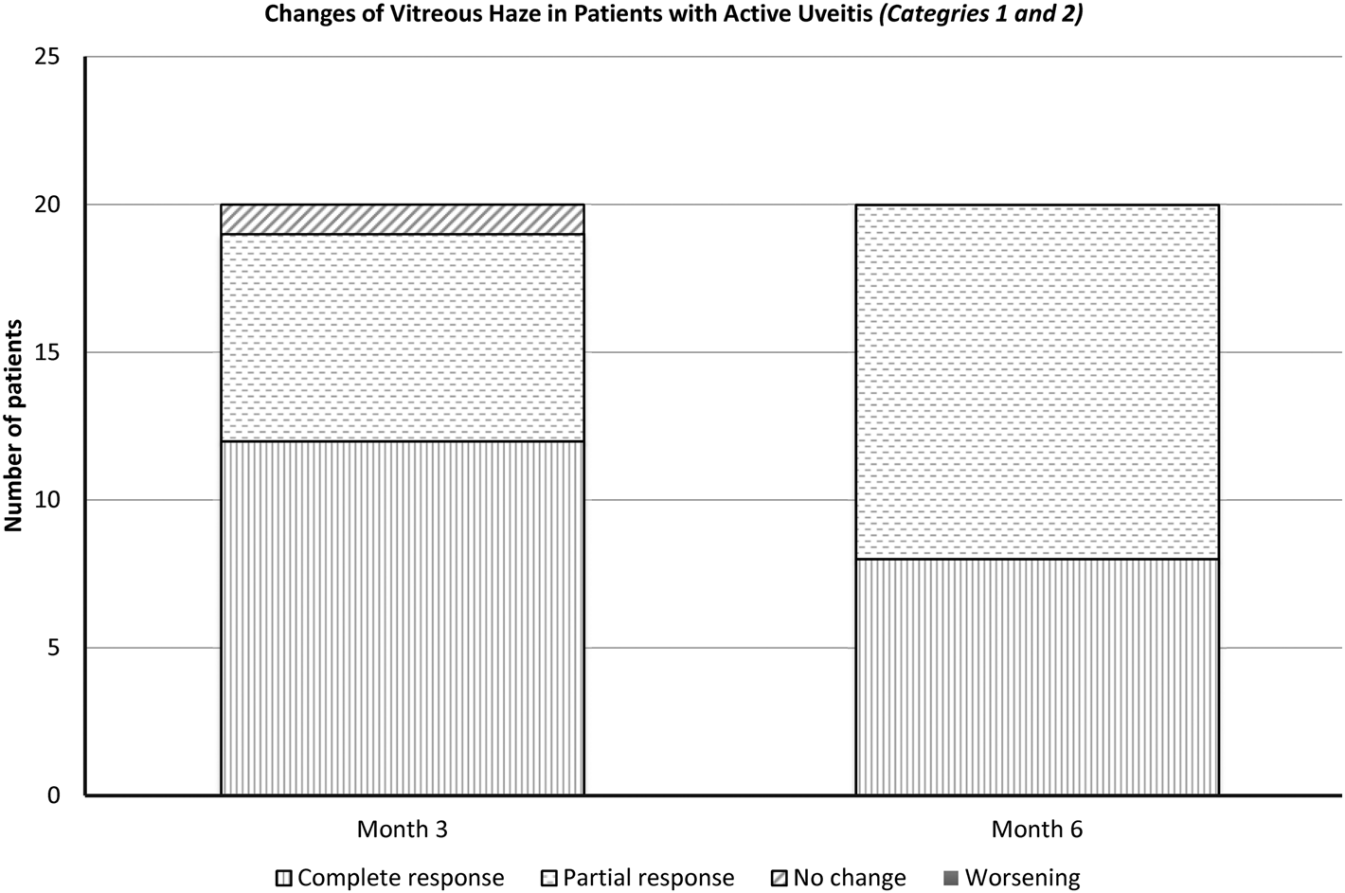
Change in vitreous haze at months 3 and 6 in patients with active uveitis at baseline.

**Response to treatment** In patients with active uveitis at baseline (disease categories 1 and 2), eight subjects (40%, four from group 1 and four from group 2) achieved complete response to treatment (reduction of vitreous haze by two steps or more or reduction of one step to no haze) at the primary endpoint at month 6. Four subjects (60%) showed either no change in vitreous haze or a reduction in vitreous haze of no more than one step. No patient showed worsening of vitreous haze at month 6 in these categories (Figure 2).

In category 3 (patients with inactive uveitis at baseline), seven subjects (88%) maintained stable vitreous haze at month 6 (three from group 1 and four from group 2), with four patients demonstrating no change in vitreous haze (two from each group) and three patients (one from group 1 and two from group 2) demonstrating a reduction of one step (0 vitreous haze)*.* One patient (12%) in this category showed an increase of vitreous haze of one step at month 6 (group 2).

**Corticosteroid sparing effect** Twenty subjects were receiving systemic CS at baseline, 13 in category 2 (prednisone ≥10 mg/day) and 7 in category 3 (prednisone <10 mg/day and/or IMT). Prior to screening, 7 subjects were receiving immunosuppressants other than CS (IMT); two subjects were receiving their IMT in conjunction with a prednisone dose <10 mg/day, four subjects were receiving IMT in conjunction with prednisone ≥10 mg/day, and one subject was receiving IMT as a maintenance monotherapy.

**Figure 3:**
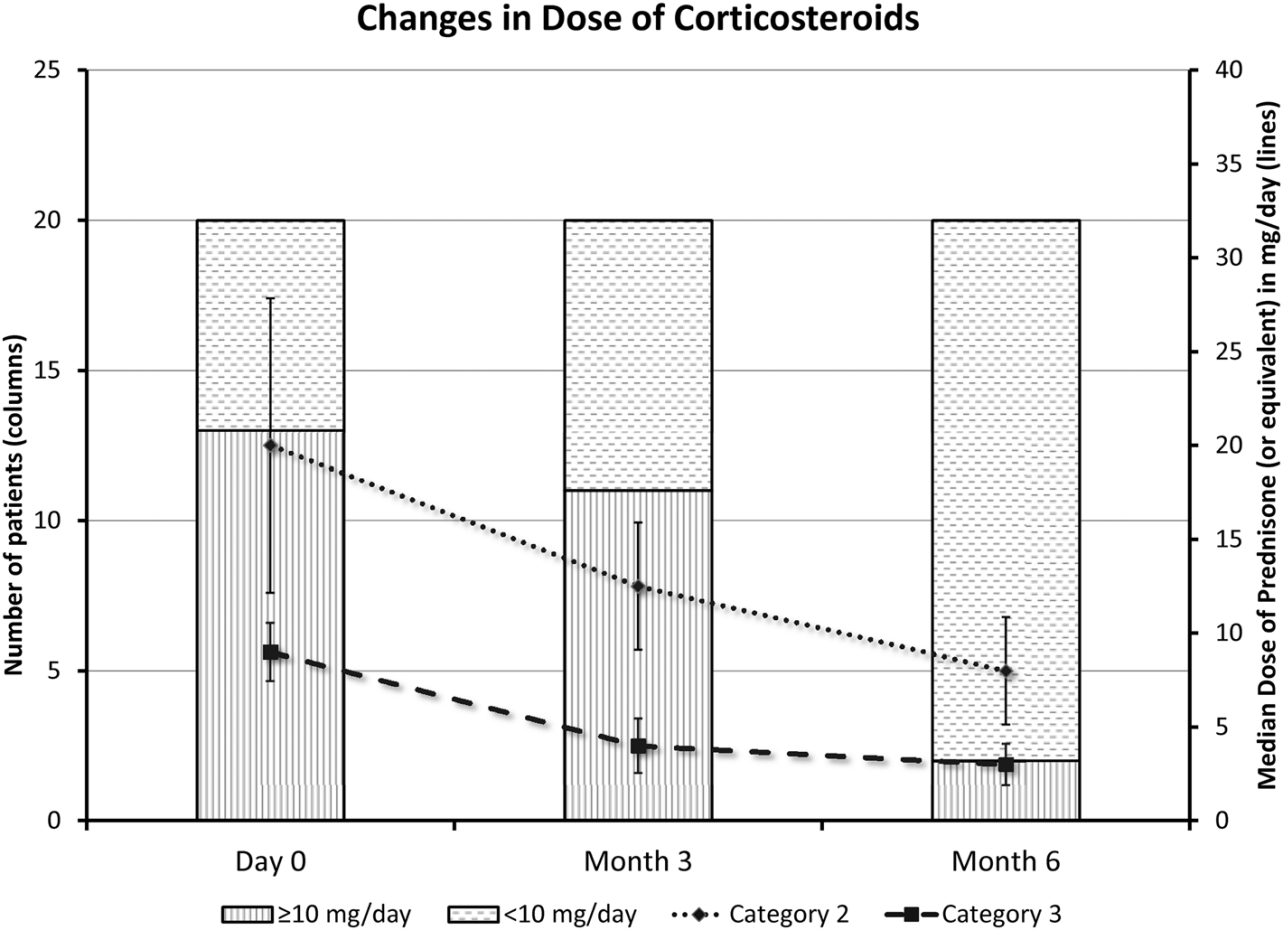
Changes in dose of corticosteroids maintained by the patients at month 3 and month 6.

Table 2 and Figure 3 demonstrate the changes in the median dose of CS among the study group and categories. In category 2, the median dose was 20 mg/day and was reduced to 12.5 mg/day at month 3 and to 8 mg/day at month 6. The dose was reduced to less than 10 mg/day in two patients (one from each group) before month 3, and by month 6, the CS dose was successfully reduced to less than 10 mg/day in 11 subjects (six from group 1 and five from group 2). It was not possible to reduce the CS dose to less than 10 mg/day in two patients (both in group 1). The CS was reduced, however, from 50 mg/day at baseline to 15 mg/day at month 6 in one patient and from 30 mg/day to 25 mg/day in the other (the dose was initially reduced to 10 mg/day at day 74; however, it was raised to 30 mg/day in the peri-operative period of bilateral combined cataract extractions and Ahmed valve implantation)*.*

The median dose of CS in category 3 was reduced from 9 mg/day at baseline to 4 and 3 mg/day at months 3 and 6, respectively.

**Changes in visual acuity** The average visual acuity (VA) at baseline was 69 (±17.7) letters (equivalent to 20/40). The average VA was 62 (±13) letters, 70 (±17) letters, and 72 (±23) letters in categories 1, 2, and 3, respectively (Table 2). At month 3, ten subjects (36%) gained one or more lines of VA (three in group 1 and five in group 2). Of the ten patients, four patients gained two lines, and one patient gained three lines (all in group 2). Four patients (14%) lost one line of VA at month 3 (two in each group). Fourteen subjects (50%) did not show any changes in VA at month 3.

At month 6, 11 subjects (39%) gained one or more lines of VA (five in group 1 and six in group 2). Of the 11 patients, 3 gained two lines (all in group 2) and 1 gained 3 lines (group 1). Six patients (21%) lost one or more lines at month 6 (three in each group) with three patients losing two lines (one in group 1 and two in group 2) and with one patient losing three lines of VA (group 2). Eleven subjects (39%) did not show any changes in VA at month 6.

**Figure 4:**
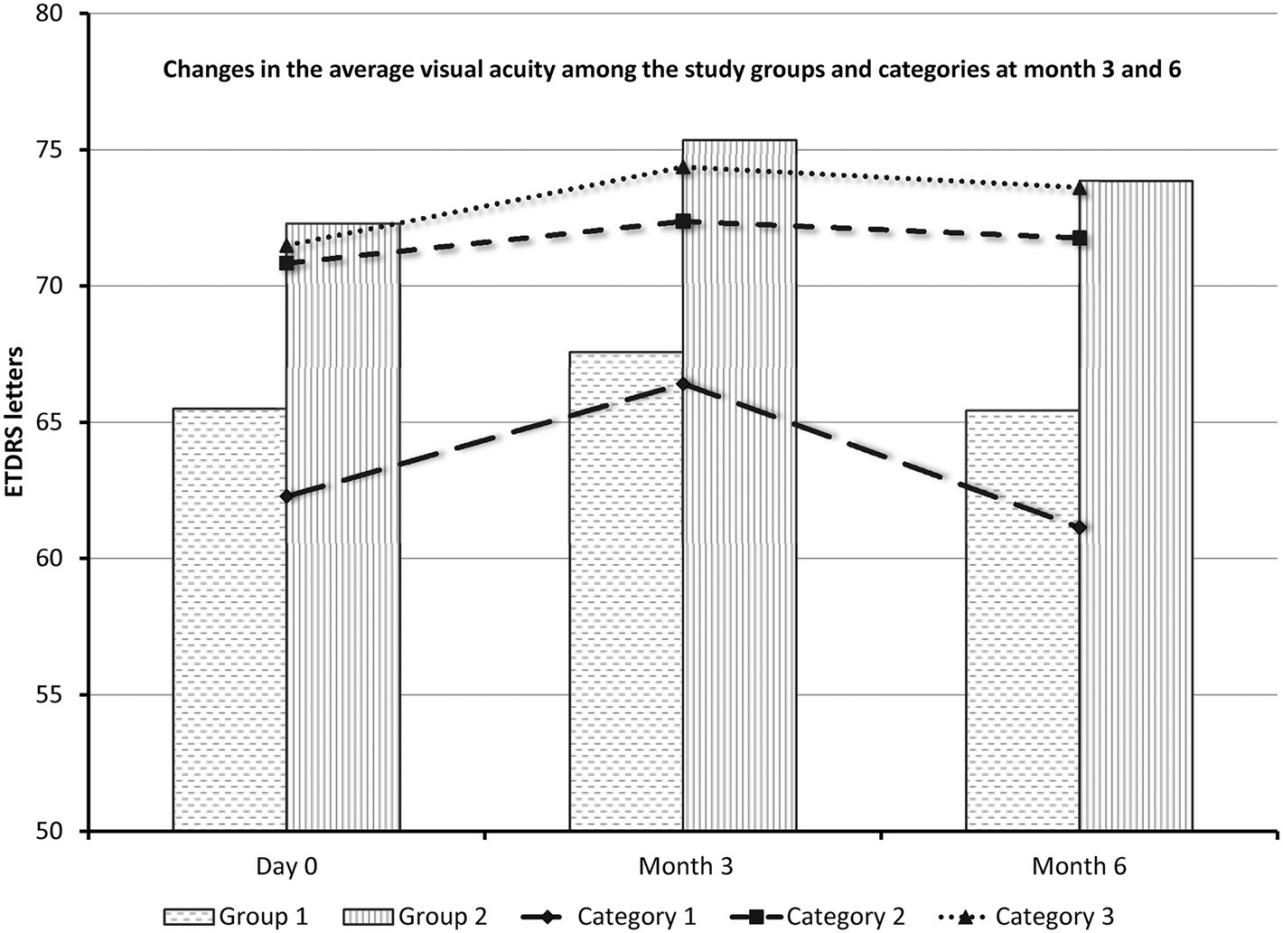
**Changes in mean visual acuity among different categories of study groups at months 3 and 6.** ETDRS, early treatment diabetic retinopathy study.

In all study groups and categories, there was a trend in gain of VA at month 3 that was maintained at month 6 in all categories of injection groups except in patients in category 1 (active uveitis at baseline without treatment), where the initial gain of VA at month 3 was lost or even reversed at month 6 (Figure 4).

**Central macular thickness** At baseline, 37% of subjects had macular edema (*n* = 11, seven in group 1 and four in group 2), with an average central macular thickness (CMT) of 505 μm (±156) on spectral domain optical coherence tomography (OCT). CMT in patients without macular edema (*n* = 17) did not show changes from baseline in any patient, either at month 3 or at month 6, with an average thickness of 272 μm (±27 μm), 266 μm (±27 μm), and 265 μm (±29 μm) at baseline, month 3, and month 6, respectively.

**Figure 5:**
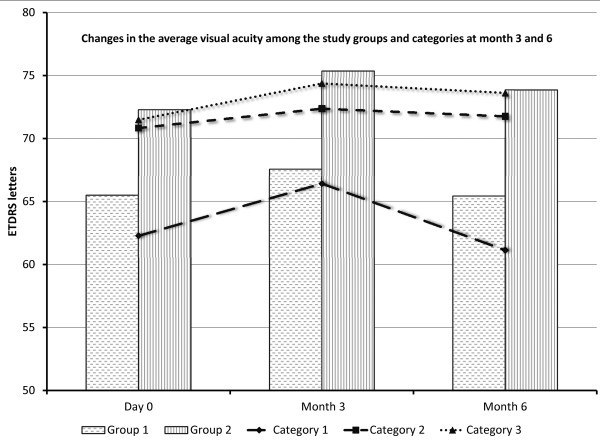
**Changes in mean central macular thickness from baseline.** Patients with macular edema at months 3 and 6 in different study groups.

In patients with ME at baseline, CMT decreased in group 1 from an average of 510 μm (±194 μm) at baseline to 454 μm (±186 μm) at month 3, then increased to 615 μm (±168 μm) at month 6 (Figure 5), a mean change of −75 and 105 μm at months 3 and 6, respectively. Group 2 showed reduction of CMT from 481 μm (±131 μm) at baseline to 448 μm (±74 μm) at month 3 and 451 μm (±114 μm) at month 6, a mean change of −33 and −30 μm at months 3 and 6, respectively.

**Figure 6:**
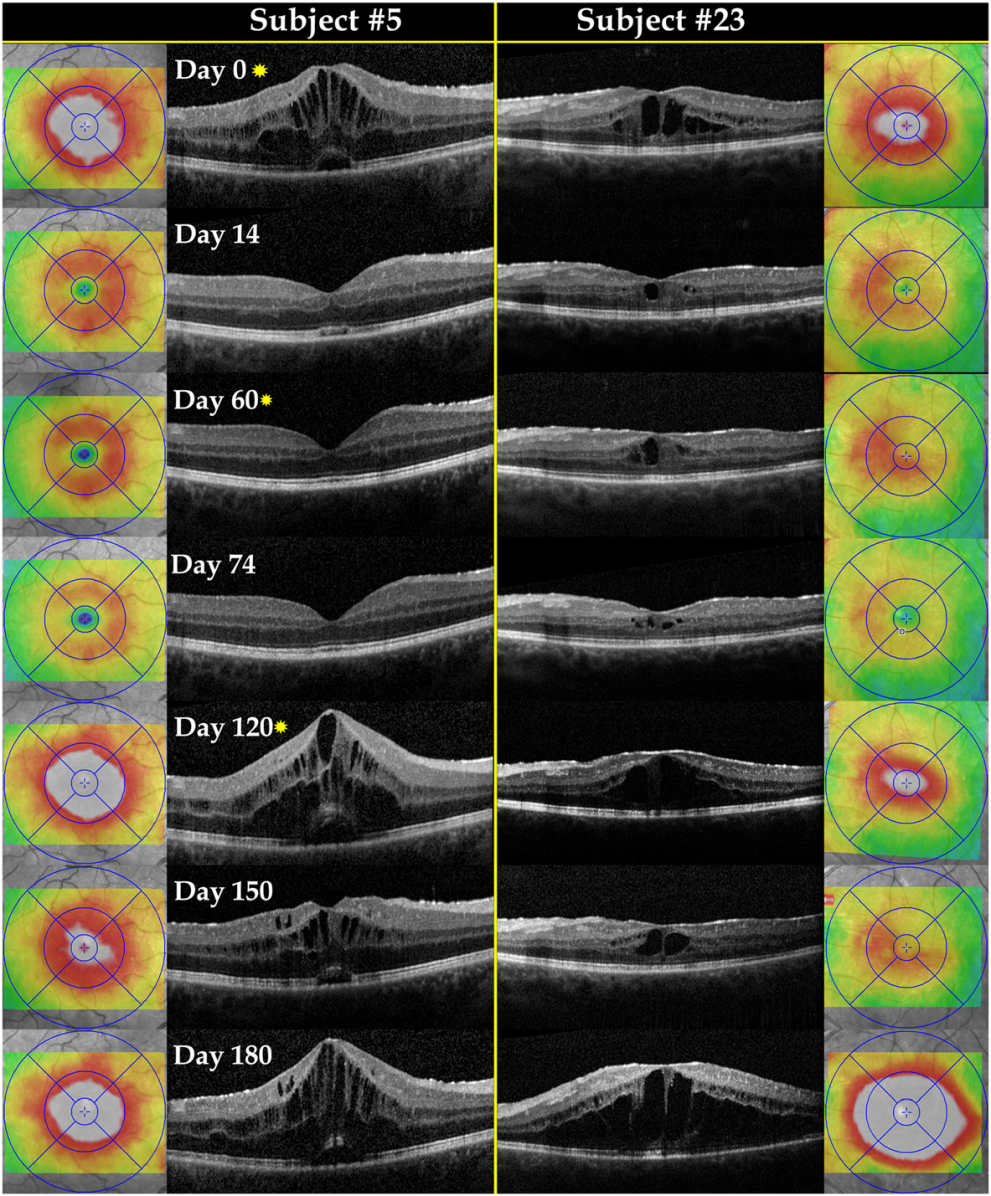
**Spectral domain optical coherence tomography horizontal scans and thickness maps at various study time points.** Two study subjects presented with macular edema at baseline. Both patients had posterior uveitis at baseline and were randomized to group 2 (subconjunctival group). At baseline, subject 5 had active uveitis and was receiving no systemic immunosuppressants (study category 1). Subject 23 had active uveitis at baseline and was receiving prednisone 20 mg/day (study category 2); the prednisone dose was tapered to 15 mg/day at month 3 and to 7.5 mg/day at month 6. Sirolimus was injected at days 0, 60, and 120 (yellow asterisks). Noticeable reduction in macular edema and central macular thickness was consistently observed 14 to 30 days following the injection with diminished response observed in the visits following day 120.

At month 3, six patients (three in group 1 and two in group 2) showed significant reduction of CMT with two patients (both in group 1) showing complete resolution of macular edema (Figure 6)*.* However, at month 6, only two patients continued to show reduction in CMT; CMT increased to its baseline level in two other patients and significantly increased in the last two (both had complete resolution at month 3; Figure 6)*.*

Two patients showed steady increase in CMT at both month 3 and month 6, and three patients did not show significant changes in CMT either at month 3 or month 6.

**Fellow eyes** At baseline, 12 fellow eyes had active uveitis (four in category 1 (33%) and eight in category 2 (67%)); 16 had inactive uveitis. At the primary endpoint at month 6, 79% (19/28) of the fellow eyes received at least one sirolimus injection, subconjunctival or intravitreal (with a total of 42 injections); 7 fellow eyes (44%), which were inactive at baseline, had flare-ups of uveitis that were treated with sirolimus during the course of the study with tapering of the CS. Thirty-eight percent (16/42) of the injections to the fellow eyes were administered during the period from day 0 to month 2, 36% (15) between months 2 and 4, and 28% (11) between months 4 and 6.

None of the fellow eyes received any other form of intra- or peri-ocular injections other than sirolimus. During the specified period, the fellow eyes received 12 injections in category 1 (1.7 injections per patient), 13 injections in category 2 (1 injection per patient), and 15 injections in category 3 (1.9 injections per patient).

**Quality of life** Mean scores were calculated for each of the subcategories and for total score at baseline and month 6 (Table 3). The change in the overall visual function questionnaire (VFQ) score demonstrated statistical significance at month 6 compared to baseline (*p* < 0.05). Six of 12 subcategories demonstrated significant improvements in their mean VFQ scores at month 6 compared to baseline. There was a statistically significant improvement in the general vision, ocular pain, distance activities, visual mental health, visual role difficulties, and visual dependency subcategories.

**Table 3:** VFQ-39 subcategory scores and mean total score stratified by date of examination

**VFQ subcategory**	**Baseline**	**Month 6**	**Difference**	***P *value**
General health	66.20	68.80	2.59	0.428
General vision	63.52	70.37	6.85	0.010
Ocular pain	70.83	81.94	11.11	0.013
Near activities	73.21	74.55	1.34	0.607
Distance activities	75.43	81.48	6.05	0.026
VS social functioning	87.35	91.98	4.63	0.100
VS mental health	57.50	69.26	11.76	0.000
VS role difficulties	69.21	76.39	7.18	0.035
VS dependency	79.40	88.19	8.80	0.045
Driving	70.31	74.65	4.34	0.069
Color vision	90.74	95.37	4.63	0.232
Peripheral vision	78.70	83.33	4.63	0.289
Total VFQ score	72.33	79.44	7.11	0.001

*P* values were calculated using Student's *t* test. VS, versus*.*

Mean total VFQ scores were calculated at baseline and month 6 and stratified by disease activity (Table 4). There was a significant improvement in mean VFQ score in the subconjunctival group. The intravitreal injection group demonstrated an improvement in VFQ scores; however, the results had borderline significance (*p* = 0.09). Subjects in category 1 had lower baseline scores than the other two groups. Although this group demonstrated an 11.28 point improvement in score, the results had borderline significance (*p* = 0.057). Subjects in category 2 demonstrated a significant improvement in scores (*p* = 0.027), while category 3 demonstrated non-significant improvement in score (*p* = 0.221).

**Table 4:** VFQ-39 total scores stratified by visits, treatment type, and disease activity

	**Baseline**	**Month 6**	**Difference**	***P *value**
Treatment type				
Group 1	73.59	78.08	4.49	0.090
Group 2	71.16	80.71	9.54	0.005
Disease activity				
Category 1	57.95	69.24	11.28	0.057
Category 2	77.09	83.78	6.69	0.027
Category 3	77.77	81.87	4.09	0.2209

*P* values were calculated using paired *t* test.

## Discussion

Through its mechanisms of action, sirolimus inhibits the production, signaling, and activity of many growth factors and antibodies relevant to uveitis by a mechanism that is distinct from that of other immunosuppressants. Sirolimus has also been shown to downregulate the expression of many genes related to inflammation such as interleukin-8, endothelial monocyte-activating polypeptide II, granulocyte chemotactic protein 2, cyclooxygenase 1 and 2, and inducible nitric oxide synthase [20-23].

IMT has been shown to be useful in the management of uveitis while reducing the need for CS. Despite the apparent usefulness of systemic IMT in the management of uveitis, bone marrow suppression, neurotoxicity, nephrotoxicity, hepatitis, pneumonitis, diarrhea, infertility, and secondary malignancy are potential side effects that limit the use of such agents and require individualization and close monitoring of IMT [9].

Shanmuganathan and colleagues evaluated systemic sirolimus as an alternative treatment for severe non-infectious uveitis refractory to other drugs or requiring local injections or high doses of systemic CS. Sirolimus was effective as a corticosteroid-sparing drug in five of eight patients; in the other three patients, the side effects were intolerable or the drug failed to control uveitis [24].

In a retrospective study, Phillips and Wroblewski reported a case series of eight patients with severe uveitis who were treated with oral, low-dose sirolimus (1 to 4 mg/day). Out of the eight patients, three patients showed improvement of uveitis when sirolimus was given in adjunction with oral methotrexate, and one patient showed improvement with sirolimus monotherapy. The study was called a failure after serious side effects forced discontinuation of sirolimus therapy [25].

Although the class of mTOR inhibitors seems to be relatively well tolerated and offers exciting new therapeutic opportunities in different disorders, they are accompanied also by local and systemic side effects and adverse events. Systemic use of sirolimus has been associated with mucositis, skin rashes, pulmonary toxicity, hyperglycemia, and bone marrow toxicity among other toxicities including hepatobiliary disorders [26], epidermal and dermal conditions such as squamous and basal cells carcinomas and photosensitivity [26], infections [27], and renal [27] and respiratory disorders [26,28]. The frequent adverse events of the mTOR inhibitors are hematological, especially microcytic anemia, leukopenia, and thrombocytopenia [29-33]. It was recently argued that low-dose oral sirolimus increases the risk of menstrual-cycle disturbances and ovarian cysts. It was also postulated that monitoring of sirolimus-associated ovarian toxicity is warranted and might guide clinical practice with the use of mTOR inhibitors [34]. The systemic morbidities associated with immunosuppressants, either CS or IMT, have encouraged the conduct of clinical trials that aimed at the development of local therapies that can be used in controlling uveitis while minimizing the potential side effects. In a 3-year multicenter clinical trial, the fluocinolone acetonide intravitreal implant showed control of uveitis with significant reduction of recurrences in the implanted eyes along with improvement or stabilization of visual acuity. The implanted eyes, however, had higher risk of increased intraocular pressure that required glaucoma filtering surgery (attributable risk of 38%) and of cataract surgeries (attributable risk of 73% on previously phakic eyes). The FA implants certainly can provide an alternative approach for prolonged control of inflammation in non-infectious uveitis. Nevertheless, its usage may not be possible in all situations because of the morbidities associated with such therapy [35]. Intravitreal formula of methotrexate was also evaluated in a prospective study for the treatment of uveitis and uveitic cystoid macular edema. Fifteen eyes from 15 patients with a unilateral exacerbation of non-infectious uveitis received single intravitreal injection of 400 μg methotrexate. The treated eye showed significant improvement of VA and reduction of the inflammatory indices in 80% of the injected eyes with reduction of the CS dose in some of the study participants who were receiving CS therapy at baseline. One third of the patients relapsed after a median of 4 months [36].

In our study, both intravitreal and subconjunctival injections of sirolimus were well tolerated. The most encountered adverse event was inflammation at the injection site manifesting as conjunctival hyperemia and chemosis in patients who received sirolimus subconjunctivally. Such result is consistent with recent reports on subconjunctival injection of sirolimus, including those reported when locally administered sirolimus was investigated in diabetic macular edema [18,37]. The development of cataract in two study eyes and one fellow eye in the intravitreal group was considered incidental or secondary to the progression of the primary disease and not necessarily related to the study drug. In the first cataract case, there was a suspicion that the cataract was the result of a traumatic effect of the injection procedure; however, no track marks were observed at the back of the lens. The other two cataract surgery incidents occurred in a single patient who had a pre-existing cataract and glaucoma. It is difficult to ascertain if sirolimus has contributed to the progression of cataract and glaucoma in this particular patient, especially in the fellow eye, which already had cataract and glaucoma refractory to maximal medical therapy. Other than these two patients, no other patients developed new cataract or had significant worsening of a preexisting one. Previous studies of intravitreal [18] and subconjunctival [18,37] injection of sirolimus also did not show significant changes in the lens condition, and there were no reports of required cataract surgeries [18,37].

Although systemic adverse events were infrequent in our study, it is important to mention that identifying adverse events during the course of a clinical trial might be subjected to several pitfalls and biases. In our study, the small study sample, short follow-up period, under representation of some populations, and lack of extensive laboratory and systemic assessments may have limited our ability to detect some systemic adverse events that may have occurred. On the other hand, despite local therapy is generally preferred, the frequent clinic visits to deliver treatment, the risks associated with intravitreal injections such as sight-threatening endophthalmitis, the necessity to treat both eyes separately in cases of bilateral uveitis, which may further increase the frequency of clinic visits if both eyes are to be injected in separate sessions, and the absence of systemic benefits in patients with extra-ocular manifestations of autoimmune disease are all limitations that should not be overlooked while calculating the risk/benefit ratio of locally delivered drugs for management of non-infectious uveitis.

In the SAVE Study, the first study to evaluate subconjunctival and intravitreal delivery of sirolimus for intermediate, posterior, or panuveitis, 40% of patients with active uveitis at baseline (categories 1 and 2) showed improvement of two steps or more of vitreous haze, as measured using the Standardized Uveitis Nomenclature (SUN) working group criteria, and 60% of patients showed either no change from baseline or one-step reduction of vitreous haze. The reduction in vitreous haze at the primary endpoint was statistically significant in both study groups (*p* < 0.5). In this study, no significant differences in response profile were detected based on route of delivery, i.e., both study groups were equally responsive to treatment. The improvement in the inflammatory indices in category 2 was associated with reduction of the adjunct corticosteroid dose in all patients (*n* = 13) with the majority (85%) of patients successfully tapered to less than 10 mg/day of CS at month 6. The improvement in the inflammatory indices of category 1 was achieved without the use of CS at any time point during the study. Overall, 88% of the patients with inactive uveitis at baseline (category 3) maintained the quiescence of uveitis at month 6 while the corticosteroid dose was successfully tapered in all patients with a median reduction of 6 mg/day (from 9 to 3 mg/day) by month 6. As the inclusion criteria for categories 1 and 2 did not require enrolled patients to have 2+ or more vitreous haze, not all enrolled subjects had the potential to improve two or more steps. As an exploratory study, SAVE was designed to evaluate any potential efficacy of sirolimus in uveitis, and thus allowed entry of ≥1+ vitreous haze. Nevertheless, 40% of subjects showed complete response to sirolimus injections.

In our study, about one third of participants showed improvement of VA at month 6 with half of the study participants showing visual stability and 20% losing one or more lines of VA. The VA at baseline was quite good and hence allowed lesser potential for significant visual gain. In patients who had macular edema at baseline (*n* = 11), there was initial reduction of central macular thickness at month 3 in about half of the patients (*n* = 6). However, such reduction in macular thickness at month 3 has continued at month 6 only in two patients out of the 6 and was maintained in another two. The relative worsening of macular edema at month 6, when compared to month 3, could be explained by the longer interval between treatments and measuring of the macular thickness at month 6, when compared to month 3, which implies that a higher dose and/or more frequent injections of sirolimus may be necessary to achieve and maintain satisfactory outcomes.

Patients in our study also have shown improvement of their quality of life as evidenced by the responses on VFQ-25. The visual functioning questionnaire revealed statistically significant improvement in the overall outcome (*p* < 0.5) with significant improvements in 6 of the 12 subcategories of the test. As may be expected, patients with inactive uveitis at baseline showed the least improvement (*p* = 0.22). After the discontinuation of systemic IMT for the study subjects to be enrolled in the SAVE Study and the tapering of CS, the elimination of being fatigue and suffering from adverse events has most likely contributed to the improvement in the quality of life for the patients.

## Conclusions

In conclusion, the SAVE Study has provided informative and valuable insights toward the goals of identifying effective local therapy for uveitis and ocular inflammatory diseases. Local administration of sirolimus, either intravitreally or subconjunctivally, appears to be well tolerated in patients with non-infectious uveitis. Sirolimus delivered either intravitreally or subconjunctivally has demonstrated bioactivity as an IMT and corticosteroid-sparing agent in reducing vitreous haze and cells and improving VA. Long-term outcomes, beyond 6 months, and additional phase 2 and 3 clinical trials, which are being conducted in the USA and other countries, are warranted to confirm the role of locally delivered sirolimus as an immunomodulatory therapeutic agent and to determine its appropriate dosage and frequency of treatments.

## Methods

SAVE is a proof-of-concept, open-label, randomized clinical study conducted at the Wilmer Eye Institute, Johns Hopkins University School of Medicine (Baltimore, MD, USA) to assess the safety, tolerability, and bioactivity of intravitreal and subconjunctival injections of sirolimus in patients with non-infectious uveitis. The study was approved by the Johns Hopkins University Institutional Review Board and was conducted in compliance with the Declaration of Helsinki, US Code of Federal Regulations Title 21, and the Harmonized Tripartite Guidelines for Good Clinical Practice (1996). Before screening, all the subjects involved in the SAVE Study reviewed and signed informed consent. The SAVE Study is registered at ClinicalTrials.gov (identifier: NCT00908466).

Consented patients with non-infectious intermediate, posterior, and panuveitis were screened for the study. Enrolled patients were stratified at baseline into three categories: (1) active disease and receiving no treatment, (2) active disease and receiving prednisone ≥10 mg/day (or equivalent dose of another CS) and/or at least one other systemic immunosuppressant, and (3) inactive disease and receiving prednisone <10 mg/day (or equivalent dose of another CS) and/or at least one other systemic immunosuppressant.

In the SAVE Study, active disease was defined as having at least 1+ vitreous haze, using the SUN Working Group/National Eye Institute - Nussenblatt scale [38,39]. Inactive disease was defined as having vitreous haze of 0.5+ or less and vitreous cell count of 0.5+ or less, using the SUN Working Group/National Eye Institute (NEI) scale [38,39]. All IMT agents were discontinued at least 30 days prior to the first administration of the study drug at day 0. Patients who were not receiving CS at screening were not allowed to receive any CS in the interim 30-day period prior to day 0. Systemic CS therapy at baseline was allowed to continue for patients who were already receiving CS therapy. Systemic CS was tapered immediately upon initiation of the first dose of sirolimus. For patients in category 2, the aim was to reduce the dose of CS to <10 mg/day. For patients in category 3, the aim was to discontinue CS or to reduce the dose to less than 5 mg/day.

Patients in each category were randomized in a ratio of 1:1 into one of two treatment groups; group 1 received intravitreal injections of sirolimus in a dose of 352 μg, and group 2 received subconjunctival injections of sirolimus in a dose of 1,320 μg. Three mandatory injections of either subconjunctival or intravitreal of sirolimus were given at days 0, 60, and 120. Follow-up visits were scheduled at 14 and 30 days (±2 days) after each injection. The primary endpoint of the SAVE Study was set at month 6. Patients are being monitored up to month 12. During the period from month 6 to month 12, patients with residual or recurrent uveitic activity are allowed to receive additional treatments with sirolimus up to every 2 months leading to a maximum of six injections over the 12-month study duration. Only the primary endpoint results at 6 months for the study eyes are being reported in this manuscript. The eligibility inclusion and exclusion criteria are listed in an online supplement (Additional file 1: Table S1).

### Fellow eyes

In patients with bilateral uveitis, the eye with more advanced disease was chosen as the study eye. If both eyes were equally affected, the study eye was chosen at the investigator's discretion prior to randomization. If the standard-of-care local therapies to the fellow eye were contraindicated, proved ineffective, or refused by the patient, then sirolimus injections were administered to the fellow eye at the investigator's discretion and at the same dose and route of administration of the study eye, but at least 14 days apart from the study eye injection.

### Administration of study drug

Sirolimus is formulated as clear, non-aqueous solutions in a vehicle composed of polyethylene glycol 400 (PEG 400) and ethanol (200 proof). Both PEG 400 and ethanol are widely used solubilizing excipients in injectable formulations [40]. Sirolimus was supplied frozen as 0.5 ml of sterile injectable solution in 2.0 ml vials. Once the administration route was determined for a patient, a vial of sirolimus was removed from the freezer and thawed by rotating the vial between the palms of the hands for a minimum of 5 min or by setting the vial at room temperature for a minimum of 30 min. Topical anesthesia and antiseptic measures were performed prior to injection employing standard procedures. A 30-gauge needle was used on a Hamilton glass syringe to deliver the intravitreal injections and on tuberculin syringe to deliver the subconjunctival injections. Sirolimus injections were performed within 2 hours following the removal of the drug from the freezer. Rescue therapy was allowed for all participants at any time when one or more of pre-defined rescue criteria is met (Additional file 2: Table S2)*.*

In addition to ophthalmological assessment at each study visit (for the study flow chart and procedures done at each visit, please refer to Additional file 3: Table S3), changes in quality of life were assessed using the extended VFQ-25. The extended VFQ-25 (consisted of 39 questions) was developed by the NEI to measure self-reported vision health status in patients with chronic eye disease [41]. The questionnaire assesses the effects of visual impairment on both task-oriented domains related to visual function and general health domains such as emotional well-being and social functioning. Each patient's questionnaire was converted to a scaled score between 0 (worst) and 100 (best) using the VFQ-25 Scoring Algorithm version 2000 [42]. Individual question scores were combined into the different subcategories as detailed in the Scoring Algorithm.

### Data collection and management

The Retinal Imaging Research and Reading Center (RIRRC) at the Wilmer Eye Institute served as the coordinating, data management, and reading Center for the SAVE Study. Readers in the RIRRC were masked to treatment groups.

### Study endpoints and statistical analyses

The main outcomes were the bioactivity and ocular tolerability of intravitreal and subconjunctival injection of sirolimus in the treatment of non-infectious uveitis. The primary bioactivity analysis was conducted at month 6 and was evaluated by assessing the proportion of patients achieving a complete or partial response in the study eye. Complete response was defined as reduction of vitreous haze by at least two steps when compared to baseline or reduction of a single step to no haze. Partial response was defined as improvement of vitreous haze of no more than one step. In patients with inactive disease at baseline (category 3), success (or efficacy) of treatment was assessed by the proportion of patients who maintained quiescent uveitis throughout the 6-month period of the study while tapering or discontinuing their previous CS therapy. In addition, the activity of disease in two patients with punctate inner choroidopathy, both enrolled in category 3, was also monitored by fluorescein angiography and high-resolution spectral domain OCT, as both had ≤0.5+ vitreous haze at the time of enrollment.

The secondary bioactivity endpoint was defined as the ability of sirolimus to reduce or prevent flare-up of uveitis in the study eye (as expressed by the frequency of ocular attacks during the first 6-month period) as evidenced by increase in vitreous haze and cells and anterior chamber cells when compared to previous visits. Other secondary parameters included change from baseline in best-corrected visual acuity as measured by ETDRS charts and in macular thickness as measured by spectral domain OCT.

The safety and tolerability of subconjunctival and intravitreal injection of sirolimus in patients with intermediate, posterior, and panuveitis were evaluated by assessing the incidence of systemic and ocular adverse events, study drug related adverse events, severe adverse events, and serious adverse events, through intraocular pressure measurements, physical examinations, liver function tests, and other serologic markers. Baseline demographics and disease characteristics were summarized (number and percentage for categorical measures and number, mean, standard deviation, and median for continuous measures) by treatment group and by disease category within treatment group. Non-parametric statistical tests, e.g., *Wilcoxon signed rank test and Mann–Whitney test*, were employed to assess the significance of changes from baseline in vitreous haze among the different categories of study groups at month 3 and month 6. Significance of changes from baseline in VFQ was determined using paired *t* test. The statistical analysis was run using IBM SPSS Statistical package v. 19, IBM Corporation, Armonk, NY, USA.

## Abbreviations

Ca2+: Calcium; CMT: Central macular thickness; CS: Corticosteroids; ETDRS: Early treatment diabetic retinopathy study; FA: Fluorescein angiography; FDA: Food and drug administration; IMT: Immunomodulatory therapy; IOP: Intraocular pressure; ME: Macular edema; mTOR: Mammalian target of rapamycin; NEI: National eye institute; OCT: Optical coherence tomography; PEG: Polyethylene glycol; RIRRC: Retinal imaging research and reading center; SAE: Serious adverse events; SAVE: Sirolimus as a therapeutic approach for uveitis; SCJ: Subconjunctival; SUN: Standardized uveitis nomenclature; VA: Visual acuity; VFQ-25: Visual function questionnaire.

## Competing interests

QDN serves on the Scientific Advisory Board for Santen, Inc. JN and NS are employees of Santen, Inc. The remaining authors have no conflicts of interest.

## Authors' contributions

QDN, JPD, and HAL have contributed to the concept and design of this study, have been involved in the data collection and preparation and critical revision of the manuscript, and have given final approval of the version to be published. MI, YJS, and MB have contributed in the data analysis and interpretation, have been involved in drafting and critically revising the manuscript, and have given final approval of the version to be published. AW has contributed in the data collection and critical revision of the manuscript and has given final approval of the version to be published. JY has contributed in the data analysis and interpretation, has been involved in the data collection and in drafting and critically revising the manuscript, and has given final approval of the version to be published. JN and NS have contributed to the concept and design of this study, have been involved in the critical revision of the manuscript, and have given final approval of the version to be published. OS has contributed to the data collection and critical revision of the manuscript and has given final approval of the version to be published. DVD has contributed to the concept and design of this study, has been involved in the data interpretation and critical revision of the manuscript, and has given final approval of the version to be published. All authors read and approved the final manuscript.

## Supplementary Material

Additional file 1: Table S1 Inclusion and exclusion criteria. Click here for file

Additional file 2: Table S2 Rescue criteria. Click here for file

Additional file 3: Table S3 Events and procedures during the first 6 months of the study. Click here for file
