# Supplementary material for: Ocular tolerability and efficacy of intravitreal and subconjunctival injections of sirolimus in patients with non-infectious uveitis: primary 6-month results of the SAVE Study
Source: J Ophthalmic Inflamm Infect. 2013 Feb 11;3:32. doi: 10.1186/1869-5760-3-32 (PMC3610181; doi:10.1186/1869-5760-3-32)
Supplement: Additional file 1: Table S1 — Inclusion and exclusion criteria. [file 1869-5760-3-32-S1.pdf]

Supplemental Table 1. Inclusion and Exclusion Criteria

I. Inclusion Criteria	
1. ≥18 years of age<br>2. Have diagnosis of uveitis determined by the Investigator to be non-infectious based on the patient's medical history, history of present illness, ocular examination, review of systems, physical examination, and any relevant, pertinent laboratory evaluations<br>3. Patients with active uveitis, defined as having at least 1+ Vitreous Haze and/or at least 1+ Vitreous Cell Count (SUN scale), and<br>4. are receiving no other treatment; or<br>5. are receiving prednisone ≥10 mg/day and/or at least 1 other systemic immunosuppressant<br>6. Patients with inactive disease, defined as having 0.5+ Vitreous Haze or less and a grade of 0.5+ Vitreous Cell Count or less (SUN scale), and are receiving prednisone <10 mg/day and/or at least 1 other systemic immunosuppressant<br>7. Have posterior, intermediate, or panuveitis; for panuveitis, if an anterior component is present, it must be less than the posterior component<br>8. Sufficient inflammation to require systemic treatment<br>9. Best-corrected visual acuity of 20/400 or better in both eyes	
**Exclusion Criteria**	
**II. Non-Ocular**	**III. Ocular**
1. Allergy or hypersensitivity to sirolimus or fluorescein dye<br>2. Immunosuppressive therapy within 30 days of Day 0<br>3. Patients who are receiving strong inducers of CYP3A4 and P-gp Any recent infection within 30 days of Baseline<br>4. Immunocompromised patients<br>5. History of CMV infection or clinical evidence of active CMV infection at Baseline<br>6. Malignancy in remission for less than 5 years prior to study<br>7. History of other disease, metabolic dysfunction, physical examination finding, or clinical laboratory finding giving reasonable suspicion of a disease condition that contraindicates the use of an investigational drug, might affect the interpretation of the results of the study, or renders the patient at high risk for treatment complications;<br>8. Females who are pregnant or lactating and females of child-bearing potential who are not using adequate contraceptive precautions<br>9. Sexually active males with partners of child-bearing potential who are not using adequate contraceptive precautions	1. Patients with bilateral uveitis who are receiving systemic immunomodulatory therapy for the treatment of the fellow eye and cannot be controlled with standard local therapies alone<br>2. Any significant ocular disease that could compromise vision in the study eye<br>3. Any intravitreal injections or posterior subtenon's steroids within 90 days prior to Day 0<br>4. Intraocular surgery within 90 days prior to Day 0<br>5. Capsulotomy within 30 days prior to Day 0<br>6. History of vitreoretinal surgery or scleral buckling within 90 days prior to Day 0<br>7. Any ocular surgery anticipated within the first 180 days following Day 0<br>8. Intraocular pressure ≥25 mmHg (glaucoma patients maintained on no more than 2 topical medications with IOP <25 mmHg are allowed)<br>9. Pupillary dilation inadequate for quality stereoscopic fundus photography<br>10. Media opacity that would limit clinical visualization, intravenous fluorescein angiography (IVFA), or OCT evaluation<br>11. Presence of any form of ocular malignancy<br>12. History of herpetic infection in the study eye or adnexa<br>13. Presence of known active or inactive toxoplasmosis in either eye<br>14. Ocular or periocular infection in either eye
