# Supplementary material for: Ocular tolerability and efficacy of intravitreal and subconjunctival injections of sirolimus in patients with non-infectious uveitis: primary 6-month results of the SAVE Study
Source: J Ophthalmic Inflamm Infect. 2013 Feb 11;3:32. doi: 10.1186/1869-5760-3-32 (PMC3610181; doi:10.1186/1869-5760-3-32)
Supplement: Additional file 2: Table S2 — Rescue criteria. [file 1869-5760-3-32-S2.pdf]

Supplemental Table 2. Rescue Criteria

**Rescue Criteria**
<u>Rescue therapy will be offered if one or more of the following conditions are met:</u><br>1. If the study eye shows no response for at least 2 months following the third scheduled injection of sirolimus (i.e., at Month 6).<br>2. Worsening of primary disease in the study eye at least 1 month following the third scheduled injection of sirolimus (i.e., at Month 6) as indicated by at least a 2 point increase in Vitreous Haze or Vitreous Cell Count as compared to Baseline.<br>3. If the fellow eye shows no response for at least 2 months following the last sirolimus injection in the fellow eye despite at least 1 sirolimus injection;<br>4. Worsening of primary disease in the fellow eye at least 1 month following the last sirolimus injection in the fellow eye as indicated by at least a 2 point increase in Vitreous Haze or Vitreous Cell Count and as compared to Baseline despite at least 1 sirolimus injection;<br>5. Severe deterioration of vision in either eye as indicated by at least doubling of the visual angle as compared to Baseline.
▪ *Conditions that can explain the deterioration of vision, other than the primary disease (e.g. cataract), must be excluded before rescue therapy is allowed.*<br>▪ *Rescue therapy will be offered once deterioration of vision or worsening of primary disease has been confirmed at least 1 month following the last sirolimus injection.*
