# Supplementary material for: Ocular tolerability and efficacy of intravitreal and subconjunctival injections of sirolimus in patients with non-infectious uveitis: primary 6-month results of the SAVE Study
Source: J Ophthalmic Inflamm Infect. 2013 Feb 11;3:32. doi: 10.1186/1869-5760-3-32 (PMC3610181; doi:10.1186/1869-5760-3-32)
Supplement: Additional file 3: Table S3 — Events and procedures during the first 6 months of the study. [file 1869-5760-3-32-S3.pdf]

Supplemental Table 3. Events and Procedures during the First 6 Months of the Study

Assessments	Baseline	D 0		D 14 (± 2)	D 30 (± 3)	D 60[a] (± 3)		D 74 (± 2)	D 90 (± 3)	D 120[a] (± 3)		D 134 (± 2)	D 150 (± 4)	Month 6
		Pre	Post			Pre	Post			Pre	Post			
Informed Consent	X[b]													
Confirm Patient Eligibility	X													
Medical History	X													
Ophthalmic History	X													
Concomitant Medications	X	X		X	X	X		X	X	X		X	X	X
Physical Exam and Vital Signs	X													X
NEI VFQ-25 Questionnaire		X												X
Randomization		X												
Sirolimus Injection		Inj. #1				Inj. #2				Inj. #3				
BCVA by ETDRS	X	X[c]		X	X	X		X	X	X		X	X	
Intraocular Pressure	X	X	X[d]	X	X	X	X[d]	X	X	X	X[d]	X	X	X
Slit Lamp Biomicroscopy	X	X	X[d]	X	X	X	X[d]	X	X	X	X[d]	X	X	X
Indirect Ophthalmoscopy	X	X	X[d]	X	X	X	X[d]	X	X	X	X[d]	X	X	X
Post Injection Assessment			X[d]			X	X[d]			X	X[d]			X
Fundus Photography	X	X[e]				X				X				X
Fluorescein Angiography	X	X[e]				X				X				X
Optical Coherence Tomography	X	X[e]		X	X	X		X	X	X		X	X	X
Liver Function Tests	X			X										X
Serum Pregnancy Test	X[f]													X
Urine Pregnancy Test		X[f]				X[f]				X[f]				X[f]
Adverse Events	X[g]	X	X	X	X	X	X	X	X	X	X	X	X	X

a. If a patient does not receive Injection #2 (Day 60) (due to rescue therapy prior to Injection #2 or any other reason), the patient will not receive Injection #3 (Day 120) and will not be required to be seen at Days 74 and 134. If a patient receives Injection #2 but does not receive Injection #3 (Day 120) (due to rescue therapy prior to Injection #3 or any other reason), the patient will not be required to be seen at Day 134. In addition, if a patient receives rescue therapy, they will not be eligible for treatment in the fellow eye or optional treatment at Months 6, 8, or 10 but will be required to attend all visits except Days 74 and/or 134.

b. Informed Consent Form - obtain prior to conducting any study-related activities.

c. If the patient has lost or gained <5 letters compared to the Baseline BCVA, continue with Day 0 assessments and Injection #1; if the patient has lost or gained ≥5 letters compared to the Baseline BCVA, the patient cannot receive Injection #1 and is required to be rescreened in order to participate in the study. A new Baseline visit should be scheduled and all Baseline parameters are to be repeated.

d. Days 0, 60 and 120 - perform slit lamp biomicroscopy, indirect ophthalmoscopy, and light perception within 30 minutes after sirolimus injection, and IOP 30 minutes after sirolimus injection. If IOP is increased, perform again 60 minutes after injection.

e. Based on the Investigator's discretion, OCT, fundus photography, and fluorescein angiogram may not be performed at Day 0.

f. Serum pregnancy (HCG) test to be performed at Baseline, urine pregnancy (HCG) to be done prior to Injection #1 on Day 0, prior to Injection #2 on Day 60, and prior to Injection #3 on Day 120. Urine pregnancy test is also required prior to any treatment in the fellow eye. A positive urine pregnancy test will be confirmed by a serum pregnancy test.

g. Adverse events – begin capturing once the Informed Consent Form has been signed.
